# CBP Expression Contributes to Neuropathic Pain via CREB and MeCP2 Regulation in the Spared Nerve Injury Rat Model

**DOI:** 10.3390/medicina60060989

**Published:** 2024-06-17

**Authors:** Chae-Chil Lee, Ki-Bong Park, Min Seok Kim, Young Dae Jeon

**Affiliations:** Department of Orthopedic Surgery, University of Ulsan College of Medicine, Ulsan University Hospital, Ulsan 44033, Republic of Korea; everest@uuh.ulsan.kr (C.-C.L.); kbpark@uuh.ulsan.kr (K.-B.P.); 0736045@uuh.ulsan.kr (M.S.K.)

**Keywords:** neuropathic pain, CREB-binding protein (CBP), methyl-CpG-binding protein 2 (MeCP2)

## Abstract

*Background and Objectives*: This study aimed to investigate the relationship between neuropathic pain and CREB-binding protein (CBP) and methyl-CpG-binding protein 2 (MeCP2) expression levels in a rat model with spared nerve injury (SNI). *Materials and Methods*: Rat (male Sprague-Dawley white rats) models with surgical SNI (n = 6) were prepared, and naive rats (n = 5) were used as controls. The expression levels of CBP and MeCP2 in the spinal cord and dorsal root ganglion (DRG) were compared through immunohistochemistry at 7 and 14 days after surgery. The relationship between neuropathic pain and CBP/MeCP2 was also analyzed through intrathecal siRNA administration. *Results*: SNI induced a significant increase in the number of CBPs in L4 compared with contralateral DRG as well as with naive rats. The number of MeCP2 cells in the dorsal horn on the ipsilateral side decreased significantly compared with the contralateral dorsal horn and the control group. SNI induced a significant decrease in the number of MeCP2 neurons in the L4 ipsilateral DRG compared with the contralateral DRG and naive rats. The intrathecal injection of CBP siRNA significantly inhibited mechanical allodynia induced by SNI compared with non-targeting siRNA treatment. MeCP2 siRNA injection showed no significant effect on mechanical allodynia. *Conclusions*: The results suggest that CBP and MeCP2 may play an important role in the generation of neuropathic pain following peripheral nerve injury.

## 1. Introduction

Without altering the DNA sequence, gene expression is known to be regulated by epigenetic regulation, which includes histone acetylation, DNA methylation, and non-coding RNA [1]. Epigenetic regulation in the central nervous system is closely related to neuronal-plasticity-related cranial development, control of brain function, and pathophysiology of cranial nerve diseases. Active research regarding brain developmental disorders, memory and learning, depression, drug addiction, brain damage, and degenerative neurological diseases is on-going [2,3].

Histone acetylation is regulated by histone acetyltransferase (HAT), which promotes gene expression by increasing histone acetylation, and histone deacetylase (HDAC), which reduces histone acetylation and inhibits gene expression [4]. The CREB-binding protein (CBP), which belongs to the HAT family, increases gene expression by acetylating key histone proteins related to the enhancer or promoter region of the gene to be activated [5,6]. Zhu et al. [7] identified an increase in CBP in the spinal cord (SC) of the neuropathic model with a chronic constriction injury (CCI) to the sciatic nerve through immunohistochemistry (IHC) and suggested a correlation between CBP and neuropathic pain.

When DNA is methylated by DNA methyltransferase (DNMT), methyl-CpG-binding domain proteins inhibit DNA binding of transcription factors or gene expression by specifically binding to methylated DNA; among the methyl-CpG-binding domain proteins, methyl-CpG-binding protein 2 (MeCP2) is the first cloned protein [8]. MeCP2 epigenetically causes Rett syndrome due to its gene abnormality [9], and in the central nervous system, it is mainly expressed in nerve cells [4,10]. Many studies suggest that MeCP2 is epigenetically related to the pathophysiology of pain. Géranton et al. [11] injected adjuvants into the hindfoot of rats with induced inflammatory pain. They observed an increase in both MeCP2 phosphorylation and transcription factors in the spinal cord (SC), indirectly demonstrating an association between DNA methylation and inflammatory pain control. Wang et al. [12], through IHC and Western blotting, identified an increase in MeCP2 in the SC of the neuropathic model with CCI to the sciatic nerve. Meanwhile, Géranton et al. [11] observed a decrease in the gene expression of MeCP2 and DNMT1 in the SC of the neuropathic model with spared nerve injury (SNI) using real-time reverse transcriptase polymerase chain reaction and a decrease in MeCP2 in injured neurons of the dorsal root ganglion (DRG) through IHC.

Many studies suggest that MeCP2 and CBP are related to the induction mechanism of neuropathic pain. Although CBP expression is increased in the SC of the neuropathic model [7], reports on the altered expression in the DRG are lacking. The expression of MeCP2 in the SC of the neuropathic model is not consistent in previous studies; the expression of MeCP2 in the DRG is known to decrease, but the types of neuronal cells showing the changes have not been determined previously [12,13]. This study aimed to investigate the changes in MeCP2 and CBP expression in the SC and DRG of a rat neuropathic pain model using IHC as well as to investigate the relationship between neuropathic pain and CBP/MeCP2.

## 2. Methods

### 2.1. Experimental Animals

Twelve-week-old male Sprague-Dawley white rats (250–300 g; Samtako, Osan-si, Gyeonggi-do, Republic of Korea) were used. Rats with SNI were included in the SNI group (n = 6), and naive rats were included in the naive control group (n = 5). The treatments and measurements were not randomly controlled. One author (C.-C.L.) was aware of the grouping during the allocation, conduction of the experiment, and outcome assessment. The data analysis was conducted by researchers who were blinded to this study. All the rats were healthy, had no genetic modification status, were of the same genotype, and did not have any previous procedures. The animals had an adaptation period of at least 2 weeks before the start of the experiment. Only healthy animals were used in the experiment. All the experimental animals were given enrichment activities, were free to move within a confined space both before and after procedures, and were housed socially. They were provided with solid compressed feed and tap water for nourishment and were raised in an environment maintained at 20 °C with a 12 h day/night cycle. This experimental study was approved by the local institutional Animal Care and Use Commit-tee (No. KNU2008-2-129). All applicable institutional and national guidelines for the care and use of animals were followed.

### 2.2. SNI Model Creation

Isoflurane anesthesia (2%) was administered to the rats, and the tibial and common peroneal nerves of the left sciatic nerve branches were ligated; the sural nerve was retained, and the muscle and skin were sutured [14]. The operated animals were sacrificed at 7 and 14 days. Non-operated animals were used as controls.

### 2.3. siRNA Preparation

CBP, MeCP2, and universal control siRNA were custom-made by ST Pharm (Daejeon, Republic of Korea), and the nucleotide sequences were as follows:

- CBP siRNA

Sense: 5′-CCCACAGCUAAUGGCAGCUdTdT-3′

Antisense: 5′-AGCUGCCAUUAGCUGUGGGdTdT-3′

- MeCP2 siRNA

Sense: 5′-GCUGUGAAGGAAUCUUCUA-3′

Antisense: 5′-UAGAAGAUUCCUUCACAGC-3′

- Non-targeting (NT) siRNA

Sense: 5′-AUG AAC GUG AAU UGC UCA ATT-3′

Antisense: 5′-UUG AGC AAU UCA CGU UCA UTT-3′

For the CBP siRNA treatment, a dose of 1 μg/10 μL per injection was used. This dose was administered intrathecally once daily from one day before SNI surgery to six days after surgery. The rationale for this dose design was to evaluate the effectiveness of CBP inhibition on neuropathic pain by assessing the reduction in mechanical allodynia and the expression of CBP-immunoreactive cells in the spinal cord, thereby determining the role of CBP in the development and maintenance of neuropathic pain.

For the MeCP2 siRNA treatment, two different doses were used: 1 μg/10 μL and 0.3 μg/10 μL per injection, administered intrathecally once daily from one day before to six days after SNI surgery. The rationale for using two doses was to evaluate the dose-dependent effects of MeCP2 inhibition on neuropathic pain, assessing whether different levels of suppression impact mechanical allodynia.

### 2.4. Intrathecal Injection

A 10 μL siRNA and i-Fect solution (Neuromics, Edina, MN, USA), with a 1:4 (wt/vol) ratio, or i-Fect in 2% isoflurane anesthesia was intrathecally injected directly between the 5th and 6th lumbar spines using a 27-gauge needle.

### 2.5. Measurement of Pain Response

The pain response measurement was performed between 9 am and 12 noon. Mechanical allodynia was measured by placing the experimental rat in a transparent plastic pail on the bottom of a metal mesh and inducing the paw-withdrawal response through the application of a von Frey filament (Stoeling, Wood Dale, IL, USA) vertically. The 50% paw-withdrawal threshold was measured by the up-and-down method [15].

### 2.6. IHC Method

The white rats were anesthetized, and systemic perfusion was performed with 50 mL of 0.1 M phosphate buffer (pH 7.4) and then with 500 mL of Zamboni fixative through the aorta. The L4 SC and DRG were removed, fixed in Zamboni fixative for 2 h, and placed in a 30% sucrose solution (4 °C) for 24 h. Then, the SC was placed in a phosphate-buffered saline solution and cut with a thickness of 30 μm using a freezing microtome; meanwhile, the DRG was cut to a thickness of 10 μm and attached to a glue-treated slide. After blocking with 5% normal horse serum, they were reacted with CBP antiserum (Santa Cruz Biotechnology, Santa Cruz, CA, USA; Cat. No. SC-583; dilution: 1000 times for the SC and 500 times for the DRG) and MeCP2 antiserum (Millipore, Burlington, MA, USA; Cat. No. 07-013; dilution: 500 times for the SC and 100 times for the DRG) for 24 h at 4 °C. After washing, biotinylated anti-rabbit immunoglobulin G (Vectastain Elite Kit; Vector Labs, Burlingame, CA, USA), diluted 200 times, was reacted at room temperature for 1 h, and after re-washing, the cells were reacted 250 times with a diluted avidin–biotin–peroxidase complex at room temperature for 30 min. Color development was performed with 3,3′-diaminobenzidine. After washing, cleaning, and sealing, observation was performed using an optical microscope.

### 2.7. Statistical Analyses

Statistical analysis was performed using SPSS version 12.0 (IBM Corp., Armonk, NY, USA). The measurement results of mechanical allodynia (avoidance threshold in grams) are expressed as means ± standard error. For the differences in the pain response results between the groups, the differences over each time period were analyzed by one-way analysis of variance, and the post hoc test was analyzed by Tukey’s multiple range test. Changes in the immune response of the SC and DRG stained by the IHC method were analyzed using an image analyzer (LabWorks version 4.5; UVP Inc., Upland, CA, USA). In the case of the SC, four tissue sections were selected at intervals of 200 μm in the L4 of each animal, and the number of immune response cells higher than a certain threshold observed within a rectangle of 400 × 200 μm^2^ at the dorsal horn angle was measured, and the average value was calculated. In the DRG, five tissue slices with 80 μm intervals were observed in each L4 DRG. Approximately 500 nuclei of neurons were observed, and when the immunoreactivity was higher than a certain threshold in the image analyzer, they were regarded as benign cells. In addition, neurons were classified as small (<600 μm^2^), intermediate (600–1200 μm^2^), or large (>1200 μm^2^) depending on the size of the cross-section containing the nuclei. The measurement results were expressed as mean ± standard error. Changes according to time were analyzed by one-way analysis of variance and Tukey test, and the comparison of measurements in the left and right DRGs was analyzed by paired *t*-test.

## 3. Results

### 3.1. Pain Changes in the SNI Model

**Figure 1:**
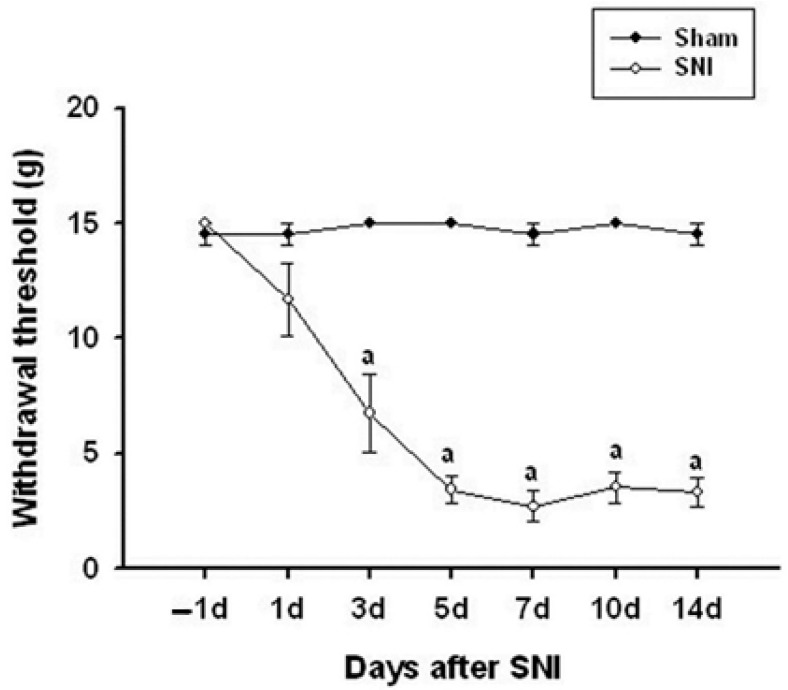
Time course of the mechanical allodynia in the ipsilateral hindfeet following spared nerve injury (SNI). Mechanical allodynia increased significantly on day 3 after SNI and persisted throughout the 14-day testing period. Data represent the mean ± standard error (n = 6 for the SNI group; n = 5 for the naive control group). ^a^ *p* < 0.05 versus naive control rats at each time point.

Mechanical allodynia, a measure of pain sensitivity, significantly increased in the SNI (spared nerve injury) model rats starting from the third day after surgery. This heightened sensitivity peaked at day 7 and remained elevated until day 14. Specifically, the 50% paw-withdrawal threshold decreased significantly, indicating increased sensitivity to mechanical stimuli. This was consistently observed in the ipsilateral hind paw, which is the side where the nerve injury was induced (Figure 1).

### 3.2. Changes in CBP Expression in the SC

**Figure 2:**
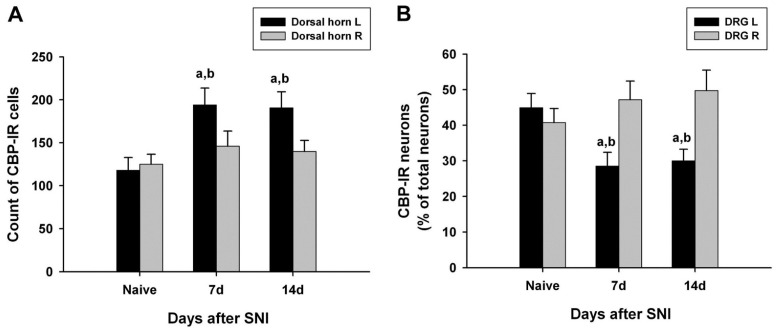
Quantified immunohistochemical data showing changes in the expression of CREB-binding protein (CBP) immunoreactivity in the ipsilateral (left, L) and contralateral (right, R) L5 spinal dorsal horns (**A**) and dorsal root ganglia (DRG) (**B**) at different time points following spared nerve injury (SNI). Data represent means ± standard error (n = 4 at each time point for the SNI groups; n = 4 for the naive control groups). ^a^ *p* < 0.05 and ^b^ *p* < 0.05 versus the naive control group and contralateral counterparts, respectively.

CBP-immunoreactive cells were observed throughout the spinal gray matter, especially in the dorsal horn. The number of CBP-immunoreactive cells in the left dorsal horn of the L4 lumbar segment on days 7 and 14 after SNI surgery was significantly increased compared with that of the normal dorsal horn of the normal and SNI groups (Figure 2A).

### 3.3. Changes in CBP Expression in the DRG

In the left L4 DRG of the control group, CBP-immunoreactive neurons accounted for 44.9%, and CBP immunoreactivity was observed mostly in small neurons (small nerve cells, 29.2%; intermediate nerve cells, 8.4%; large nerve cells, 7.4%) (Table 1). The number of CBP-immunoreactive cells in the left L4 DRG on days 7 and 14 after surgery was significantly lower than that of the right L4 DRG in the normal and SNI groups. CBP immunoreactivity was also observed most frequently in small nerve cells in the L4 DRG of the surgery group (14 days after surgery: small nerve cell, 22.5%; intermediate nerve cell, 3.6%; large nerve cell, 4.0%) (Table 1 and Figure 2B).

### 3.4. Changes in MeCP2 Expression in the SC

**Figure 3:**
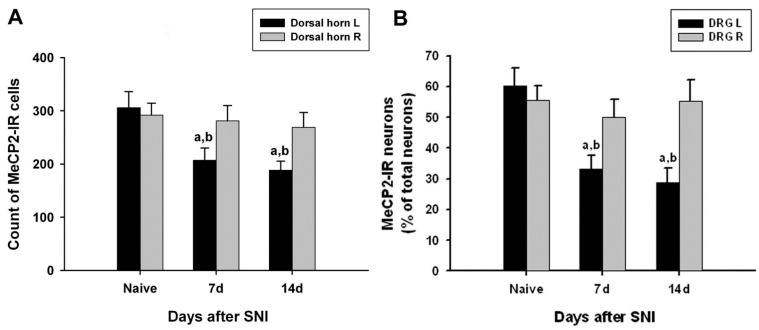
Quantified immunohistochemical data showing changes in the expression of methyl-CpG-binding protein 2 (MeCP2) immunoreactivity in the ipsilateral (left, L) and contralateral (right, R) L5 spinal dorsal horns (**A**) and dorsal root ganglia (DRG) (**B**) at different time points following spared nerve injury (SNI). Data represent mean ± standard error (n = 4 at each time point for the SNI groups; n = 4 for the naive control groups). ^a^ *p* < 0.05 versus the naive control group; ^b^ *p* < 0.05 versus the contralateral counterparts.

The number of MeCP2-immunoreactive cells in the left dorsal horn of the L4 lumbar segment on days 7 and 14 after SNI surgery was significantly lower than that of the right dorsal horn of the normal and SNI groups (Figure 3A).

### 3.5. Changes in MeCP2 Expression in the DRG

In the left L4 DRG of the control group, MeCP2-immunoreactive neurons accounted for 60.4%, and MeCP2 immunoreactivity was most commonly observed in small nerve cells (small nerve cell, 41.6%; intermediate nerve cell, 10.5%; large nerve cell, 8.2%). The number of MeCP2-immunoreactive cells in the left L4 DRG on days 7 and 14 after surgery was significantly lower than that of the right L4 DRG in the normal and SNI groups. In the L4 DRG of the surgery group, MeCP2 immunoreactivity was most frequently observed in small nerve cells (14 days after surgery: small nerve cell, 21.7%; intermediate nerve cell, 3.3%; large nerve cell, 3.7%) (Table 1 and Figure 3B).

### 3.6. Changes in Pain Response after CBP siRNA Injection

**Figure 4:**
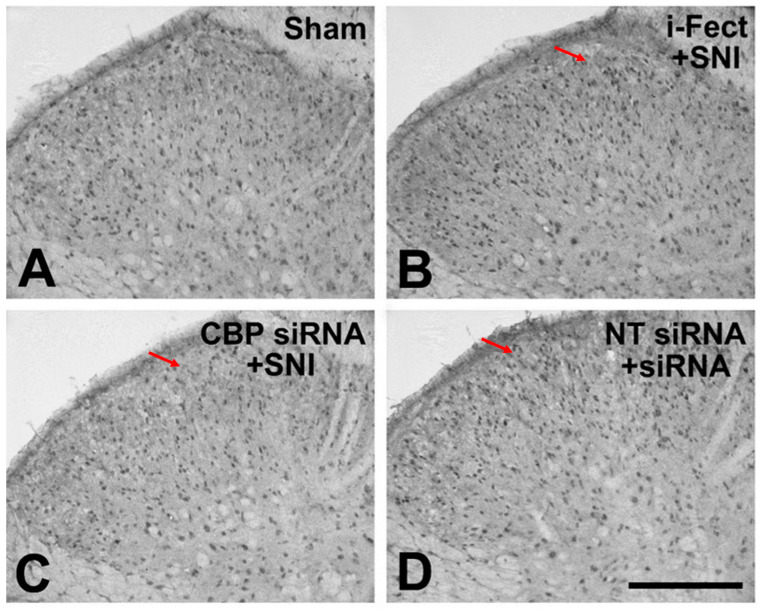
3,3′-Diaminobenzidine (DAB)-stained slides showing the effect of CREB-binding protein (CBP) siRNA treatment on increased numbers of CBP-immunoreactive cells 7 days after spared nerve injury (SNI). An increase in CBP expression following SNI was reduced in the ipsilateral dorsal horn with CBP siRNA treatment. Magnification is shown with the scale bar, which is equivalent to 200 μm. Red arrow: CBP expression, (**A**) Sham operation, (**B**) i-Fect injection group, (**C**) CBP siRNA injection group, (**D**) NT siRNA injection group.

**Figure 5:**
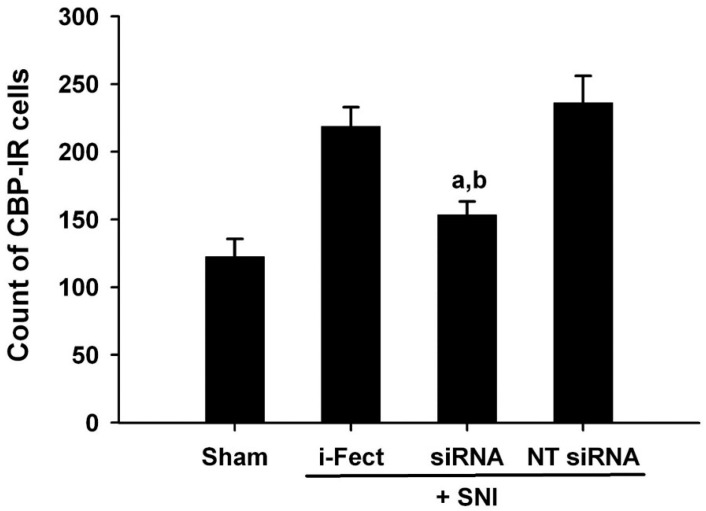
Effect of CREB-binding protein (CBP) siRNA treatment on increased CBP expression in the ipsilateral dorsal horn 7 days after spared nerve injury (SNI). Immunohistochemical analysis shows an increased numbers of CBP-immunoreactive cells expression after SNI and a reduction in these numbers with intrathecal CBP siRNA treatment. Data represent mean ± standard error (n = 4 for each group). ^a^ *p* < 0.05 versus the sham control group; ^b^ *p* < 0.05 versus the non-targeting (NT) siRNA-treated SNI group.

**Figure 6:**
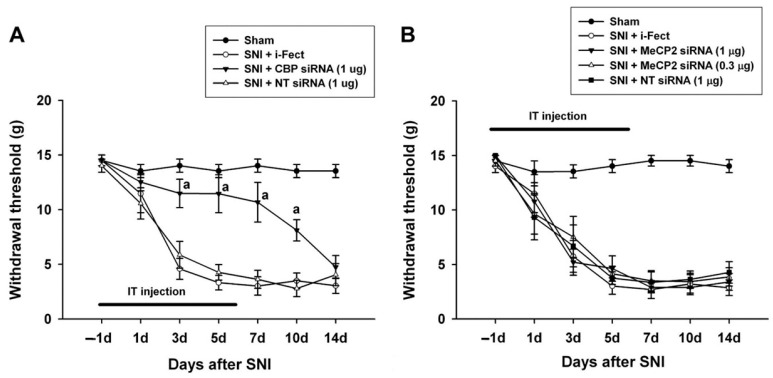
Effect of CREB-binding protein (CBP) and methyl-CpG-binding protein 2 (MeCP2) siRNA treatment on pain behaviors induced by spared nerve injury (SNI). (**A**) Intrathecal treatment with CBP siRNA applied 1 day before SNI and then daily for 7 days attenuated SNI-induced mechanical allodynia. (**B**) MeCP2 siRNA treatment had no effect on SNI-induced mechanical allodynia. ^a^ *p* < 0.05 compared with the non-targeting (NT) siRNA-treated SNI group (n = 5–6 for each group).

The effect of CBP siRNA injected in the spinal column of the neuropathic pain model on the expression of CBP in the SC was measured by IHC. When CBP siRNA (1 μg/10 μL per injection) was injected once a day from 1 day before SNI to 6 days after surgery, on day 7, i-Fect or NT siRNA in the SC was effectively reduced in the number of CBP-immunoreactive cells compared with the injection group (Figure 4 and Figure 5); in addition, mechanical allodynia was significantly inhibited in this injection group from 3 to 10 days after surgery compared with the NT siRNA injection group. Thus, CBP siRNA inhibited SNP-induced neuropathic pain (Figure 6A).

### 3.7. Changes in Pain Response after MeCP2 siRNA Injection

When MeCP2 siRNA (1 μg/10 μL or 0.3 μg/10 μL per injection) was injected once daily from 1 day before SNI to 6 days after surgery, there was no change in mechanical allodynia compared with the i-Fect or NT siRNA injection groups (Figure 6B).

## 4. Discussion

In previous studies on epigenetics-related pain syndrome, in the case of inflammatory pain, Géranton et al. [11] observed an increase in MeCP2 phosphorylation and transcription factor in the SC of white rats injected with adjuvant on the hindfoot. Chiechio et al. [16] reported that analgesic effects were obtained after subcutaneous injection of HDAC inhibitor into mice injected with formalin on the hindfoot, and acetylation of non-histone NF-κB p65 and increased expression of metabotropic glutamate receptor 2 in the DRG were also reported. Bai et al. [17] presented that, while class IIa HDACs are increased in the SC of inflammatory pain models, the class I HDACs do not change; in addition, intrathecal injection of valproic acid or 4-PB-inhibiting class I and II HDACs resulted in pain relief, but class I HDAC-inhibiting MS-275 did not exhibit analgesic effects, suggesting that class II HDAC may be involved in inflammatory pain. Furthermore, recently, a reduction in methylation in the promoter of cystathionine-*b*-synthetase gene related to pain-induced DRG in inflammatory pain models has been reported [18]. Meanwhile, in the nucleus raphe magnus, which plays an important role in the down-system pain suppression system, increased histone acetylation of the glutamic acid decarboxylase 65 (*GAD65*) gene promoter, a γ-aminobutyric acid synthase, increases the synthesis of *GAD65*, leading to analgesic effects [19].

In the case of neuropathic pain models that damage peripheral nerves, in addition to decreased expression of the Nav1.8 sodium channel and the μ-opioid receptor in the DRG, increased neuron-restrictive silencer elements and decreased histone acetylation in promoters of these genes have been reported [20]. Kiguchi et al. [21] observed that increased expression of macrophage inflammatory protein 2 and C-X-C chemokine receptor type 2, which cause neuropathic pain in neutrophils and macrophages accumulated in damaged sciatic nerve, is associated with increased acetylation of histone H3 in the promoters of these genes. It was also reported that increased expression of the monocyte chemotactic protein 3 gene, which is associated with the induction of neuropathic pain after sciatic nerve injury, is caused by decreased trimethylation of this gene promoter [22].

In this study, CBP was significantly decreased in the L4 dorsal horn and DRG at 7 and 14 days after SNI surgery compared with the control group, and MeCP2 also showed significant decrease. In the DRG of the control and SNI groups, MeCP2 or CBP was mainly expressed in small cells with C fibers. In this case, further experiments are required to determine whether these cells are peptide-expressing trkA-expressing cells or non-peptide-type IB4-binding cells. In addition, whether the decrease in MeCP2 has a role in the gene expression changes of many substances (e.g., neuropeptide Y) [23] that increase in the DRG after peripheral nerve injury as well as whether a decrease in CBP is involved in changes in gene expression of many decreasing substances (e.g., potassium voltage-gated channel) should also be investigated [23].

Mechanical allodynia decreased after CBP siRNA injection from 1 day to 6 days after SNI surgery and further decreased with CBP siRNA injection from 7 to 12 days after SNI surgery, indicating that CBP is involved in both the development and maintenance of neuropathic pain.

Wang et al. [12] reported an increase in MeCP2 in the SC of the chronic constriction injury model, whereas Géranton et al. [13] reported a decrease in MeCP2 mRNA in the SC of the SNI model; meanwhile, MeCP2 decreased in this study. This difference from the results of Wang et al. [12] appears to be due in part to the differences in the specificity of the MeCP2 antiserum used in the experiment; the MeCP2 antiserum (Millipore; Cat. No. 07-013) used in this experiment has been tested for its specificity in experiments using *MeCP2*-gene-deficient mice [13]. As reported by Géranton et al. [13] and this study, the decrease in MeCP2 in the SC following peripheral nerve injury suggests an increased expression of many genes here; this is supported by a finding of Lacroix-Fralish et al. [24] that suggests that the majority of SC gene expression increases after 7 days of peripheral neuronectomy. In addition, Wang et al. [12] reported that injecting 5-azacytidine, an inhibitor of DNMT, into the SC of the CCI model inhibited neuropathic pain, but the inhibition of neuropathic pain was not observed on the intraspinal injection of MeCP2 siRNA in this experiment. The presence of CBP in the SC was determined to be deeply associated with an increased expression of the gene after peripheral nerve injury, as seen in the results of Zhu et al. [7] and its expression in this study. In addition, Zhu et al. [25] reported that the expression of p300, of the HAT family, in the SC after peripheral nerve injury is increased, and neuropathic pain is suppressed by inhibiting the expression of p300 by intrathecal injection of p300 siRNA. However, Denk et al. [26] reported that neuropathic pain was also inhibited by increasing acetylation through intrathecal injection of the HDAC inhibitor MS275.

According to the results of this study, CBP may play an important role in the generation of neuropathic pain following peripheral nerve injury; however, there might be no such effect of MeCP2 on neuropathic pain. There were no significant changes in neuropathic pain after the intrathecal injection of MeCP2 siRNA. In addition, similar studies have shown different results regarding MeCP2 expression in neuropathic pain [27,28]. Therefore, further studies are required to clarify the underlying molecular events in epigenetic alterations [28].

## 5. Conclusions

These findings contribute to the growing body of knowledge on the epigenetic regulation of pain, highlighting CBP and MeCP2 as a potential therapeutic target for neuropathic pain management. Future research should focus on delineating the precise molecular mechanisms through which CBP and MeCP2 influences pain pathways.

## Figures and Tables

**Table 1:** Percentage of CREB-binding protein (CBP)- and methyl-CpG-binding protein 2 (MeCP2)-immunoreactive neuron profiles of small, medium, and large neuron profiles in naive control, left (ipsilateral), and right (contralateral) L4 dorsal root ganglia 7 and 14 days after spared nerve injury (SNI).

	Size of Neuron	Naive		SNI 7 Day		SNI 14 Day	
		Left	Right	Left	Right	Left	Right
CBP	Small	29.2 ± 2.5	25.7 ± 2.6	18.7 ± 3.0	32.5 ± 4.0	22.5 ± 2.1	35.1 ± 5.4
	Medium	8.4 ± 1.2	8.90 ± 1.3	4.9 ± 0.8	7.3 ± 1.2	3.6 ± 0.8	7.8 ± 0.9
	Large	7.4 ± 0.8	6.15 ± 1.8	4.9 ± 1.3	7.4 ± 1.4	4.0 ± 1.4	6.8 ± 1.5
	Total	44.9 ± 4.0	40.7 ± 4.0	28.5 ± 3.9	47.2 ± 5.2	30.0 ± 3.2	49.8 ± 5.8
MeCP2	Small	41.6 ± 4.1	38.1 ± 4.5	24.4 ± 3.5	33.4 ± 3.7	21.7 ± 3.4	38.6 ± 5.4
	Medium	10.5 ± 1.3	10.5 ± 1.3	4.9 ± 0.8	9.7 ± 1.3	3.3 ± 0.8	7.9 ± 0.9
	Large	8.2 ± 1.3	7.0 ± 0.7	4.0 ± 0.7	7.1 ± 1.3	3.7 ± 0.9	8.8 ± 1.3
	Total	60.4 ± 5.8	55.5 ± 4.8	33.3 ± 4.4	50.1 ± 5.6	28.7 ± 4.7	55.2 ± 7.0

Numbers represent mean ± standard error.

## Data Availability

Data are available upon request to the corresponding author with reasonable cause.
